# Written distractor words influence brain activity during overt picture naming

**DOI:** 10.3389/fnhum.2014.00167

**Published:** 2014-03-24

**Authors:** Michele T. Diaz, Larson J. Hogstrom, Jie Zhuang, James T. Voyvodic, Micah A. Johnson, C. Christine Camblin

**Affiliations:** ^1^Brain Imaging and Analysis Center, School of Medicine, Duke UniversityDurham, NC, USA; ^2^Department of Psychiatry and Behavioral Sciences, School of Medicine, Duke UniversityDurham, NC, USA

**Keywords:** language, overt production, fMRI, picture-word interference, phonology, semantics

## Abstract

Language production requires multiple stages of processing (e.g., semantic retrieval, lexical selection), each of which may involve distinct brain regions. Distractor words can be combined with picture naming to examine factors that influence language production. Phonologically-related distractors have been found to speed picture naming (facilitation), while slower response times and decreased accuracy (interference) generally occur when a distractor is categorically related to the target image. However, other types of semantically-related distractors have been reported to produce a facilitative effect (e.g., associative, part-whole). The different pattern of results for different types of semantically-related distractors raises the question about how the nature of the semantic relation influences the effect of the distractor. To explore the nature of these semantic effects further, we used functional MRI to examine the influence of four types of written distractors on brain activation during overt picture naming. Distractors began with the same sound, were categorically-related, part of the object to be named, or were unrelated to the picture. Phonologically-related trials elicited greater activation than both semantic conditions (categorically-related and part-whole) in left insula and bilateral parietal cortex, regions that have been attributed to phonological aspects of production and encoding, respectively. Semantic conditions elicited greater activation than phonological trials in left posterior MTG, a region that has been linked to concept retrieval and semantic integration. Overall, the two semantic conditions did not differ substantially in their functional activation which suggests a similarity in the semantic demands and lexical competition across these two conditions.

## Introduction

Language production is a critical communicative and social aspect of daily life. However, even a simple production task like picture naming includes several distinct stages such as conceptual selection, phonological retrieval, and articulation. Picture-word interference (PWI) paradigms allow researchers to examine the influence of distractors on these different stages and have been instrumental in developing theoretical models of speech (Dell and O'seaghdha, 1991; Levelt et al., 1999). In the PWI paradigm, participants are asked to name pictures while ignoring written or auditory distractors that are presented in close temporo-spatial proximity. These distractors, relative to unrelated distractors, have a behavioral influence on response times and accuracies, and presentation parameters can be manipulated to investigate different aspects of language production.

Faster response times and higher accuracies (facilitation) have generally been found when the written or spoken distractor is phonologically related to the target picture, such as the word *appetite* presented with a picture of an apple (e.g., Posnansky and Rayner, 1977; Levelt et al., 1999). This facilitation effect has been suggested to occur at the level of phonological^1^ word-form encoding, where the activation of partially overlapping phonology from the written/spoken distractor allows for more rapid lexical access during picture naming (Starreveld and La Heij, 1996). Slower response times and lower accuracies (interference) have been reported when a written distractor word is categorically related to the target image, such as the word *screwdriver* appearing with a picture of a hammer (Rosinski, 1977; Lupker, 1979; Glaser and Dungelhoff, 1984; La Heij, 1988). However, there have been some reports that not all semantically-related distractors cause interference. In several recent studies, written or spoken distractor words that were associatively related to the target (Alario et al., 2000; Abel et al., 2009, 2012) or in a part-whole relationship (e.g., bristles-toothbrush) with the target image (Costa et al., 2005) facilitated picture naming. Costa et al. suggested that when a categorically distinct distractor is a component part of the target (such as with the part-whole distractor) the semantic system can more easily activate the target concept, but due to the category differences between target and distractor the non-relevant concept is easily discarded before lexicalization. An alternative account suggests that not all words become active during naming, in particular those that may be related to the target but belong to a different category (e.g., bumper-car, La Heij et al., 2006). The extent to which these types of semantically-related distractors can facilitate or inhibit picture naming, as well as the brain regions involved in such processes, remains unclear.

In addition to behavioral studies, a limited number of experiments have examined the neural bases of phonological and semantic influences on naming. Combining PWI paradigms with fMRI allows for the identification of regions involved in semantic and phonological retrieval and can give insight into the mechanisms of facilitation and inhibition during language production. In a study examining the neural correlates of the semantic inhibition effect, de Zubicaray and colleagues found that categorically-related written distractors elicited increased activation in bilateral middle temporal gyrus (MTG) and left superior temporal gyrus (STG, De Zubicaray et al., 2001). Meta analyses and theoretical accounts (Levelt et al., 1999; Indefrey and Levelt, 2004) suggest that the activation increases in the middle portion of the left MTG and the posterior section of the left STG correspond to greater competition at the conceptual and phonological level, respectively. Activity in left inferior frontal gyrus (IFG) has been linked to semantic inhibition. For example, Abel et al. (2009, 2012) found greater activity in the pars orbitalis during categorically-related spoken distractors; this increase may reflect increased semantic competition as the IFG has been implicated when processing semantic relationships (Bookheimer, 2002) and during lexical selection (Schnur et al., 2009). In contrast, a more recent study from de Zubicaray and McMahon found decreased activation in left pars orbitalis and pars triangularis (IFG) for categorically-related spoken distractors (De Zubicaray and Mcmahon, 2009). Although the direction of the effect varies, these studies indicate that left IFG may play a role in semantic inhibition.

To our knowledge, only one previous neuroimaging study has examined the effects of different types of semantic relationships using the PWI task. Abel et al. (2009, 2012) used a category-related distractor condition and an associatively-related distractor condition in which spoken distractors and targets were situationally associated (e.g., banana-monkey), but did not necessarily share semantic features. Associatively related stimuli may produce facilitation because they often co-occur, and the presence of one may provide a strong cue for lexical access of the target. Similar to previous behavioral work the associatively-related condition showed naming time facilitation and not the classic semantic inhibition effect (Alario et al., 2000; Costa et al., 2005). The neuroimaging data, revealed that associatively-related distractors elicited less activity in the left pars orbitalis when compared to unrelated distractors as well as categorically-related distractors (Abel et al., 2009, 2012). Therefore, Abel et al. (2009, 2012) were able to show modulation in left pars orbitalis for the different types of semantic relationships: increases in activity associated with categorical interference and decreases in activity for facilitation related to associative distractors. This result suggests that this area may be related to the selection of semantic features.

As with the effects of semantic-relatedness, the neural correlates of the phonological facilitation effect in the PWI task have also been studied (De Zubicaray et al., 2002; Abel et al., 2009, 2012; De Zubicaray and Mcmahon, 2009). Both written (De Zubicaray et al., 2002) and spoken (De Zubicaray and Mcmahon, 2009) phonologically-related distractors have been reported to elicit significant priming effects and less activity in the left STG. In contrast, Abel et al. (2009, 2012) did not find any difference in left STG activity comparing unrelated and phonologically-related spoken distractors. Furthermore, phonologically-related distractors actually produced increased activation in bilateral STG when compared to associatively or categorically-related distractors (Abel et al., 2009, 2012). Although there has been some inconsistencies, overall these findings highlight the importance of these regions in semantic and phonological processes.

The present study utilized written distractors in a PWI task to examine how different types of distractors influenced brain activation during overt naming. Distractors were categorically related to the target, in a part-whole relationship with the target, phonologically related to the target, or unrelated to the target. Specifically, we wanted to examine how different types of semantic distractors influence naming. While categorically-related distractors have been shown to slow naming, there have been some reports of part-whole distractors providing facilitation. This is somewhat surprising as both distractors represent additional sources of semantic information that do not provide direct phonological or lexical information about the target. To our knowledge, this is the first picture naming fMRI study to specifically examine distractors with a part-whole relationship with the other conditions in the same design. Based on previous reports, we anticipated that phonologically-related distractors would facilitate naming (i.e., faster RTs and decreased functional activation in phonological regions) and categorically-related distractors would inhibit naming (i.e., slower RTs and increased functional activation in lexical regions) by providing competing lexical information. If naming benefits from the unique semantic relationships between parts and their whole, then we would expect the part-whole condition to elicit facilitation. However, if part-whole items are processed similarly to categorically-related items, by nature of not providing a direct lexical prime to the target word, then we would expect inhibition. Individual differences in naming ability may further influence patterns of functional activation, therefore we included a verbal fluency task outside of the scanner to examine the potential role of individual differences in production.

## Methods

### Participants

Sixteen healthy, right-handed, native English speaking adults participated (females = 8, mean age = 25.2, age range = 19–31). Everyone reported normal or corrected-to-normal vision, and no one reported a history of neurological or psychological disorders. Before the fMRI session each participant completed assessments to determine handedness, language history, and verbal fluency. In the verbal fluency assessment participants were asked to verbally generate as many items as possible that fit a certain condition. Participants were assessed on phonological categories (starts with F, A, and S) and on one semantic category (animals). Participants were given 60 s per condition to respond, and the total score across all categories was used for further analyses. Each participant provided informed consent and was paid for his or her participation. Procedures were approved by the Institutional Review Board of the Duke University Medical Center.

### Stimulus materials and procedure

**Figure 1:**
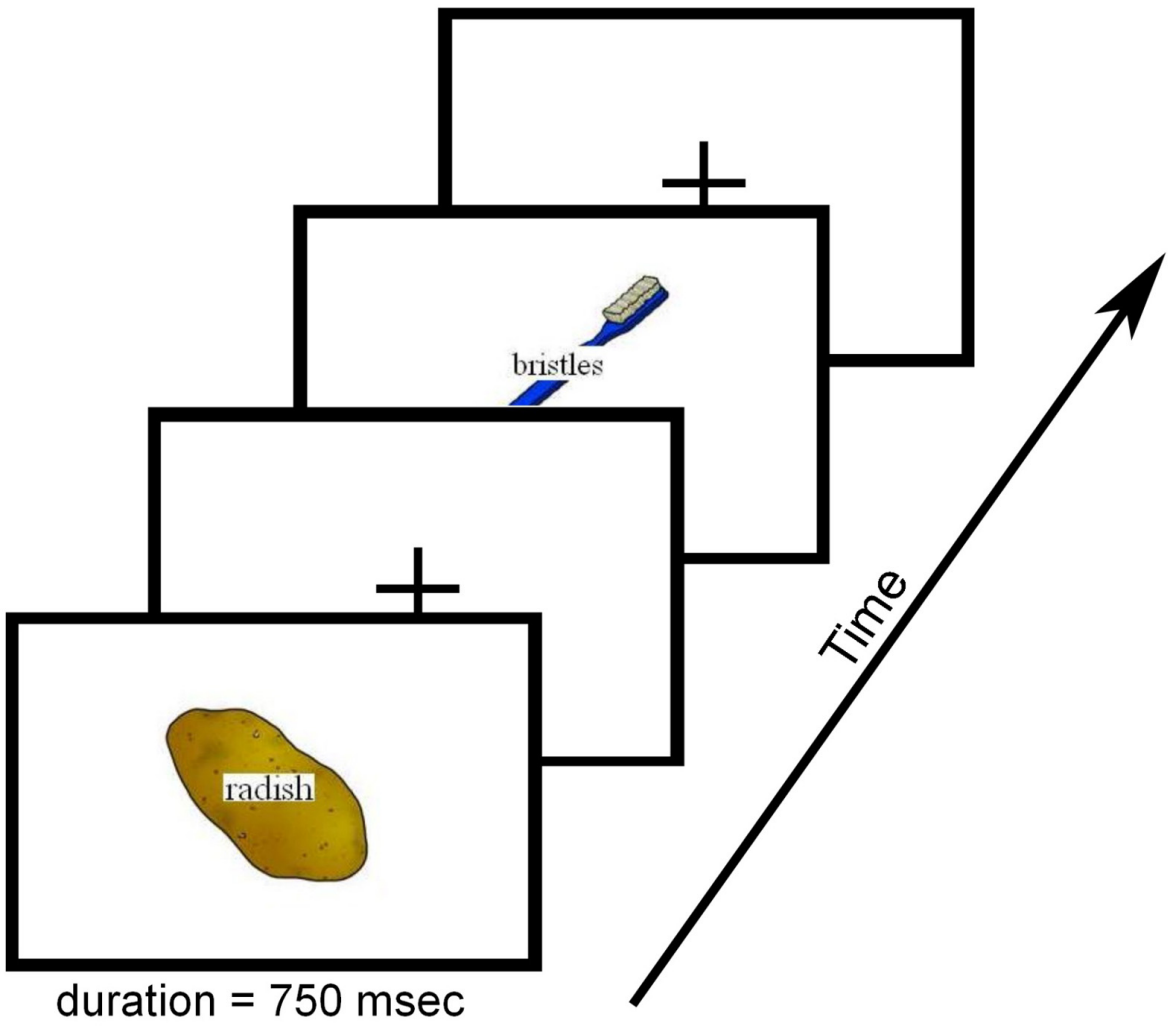
**Experimental Design**. An overview of the task design is shown here. The figure depicts a typical sequence presenting two different trial types (categorically-related and part-whole). Pictures and words were presented simultaneously (duration = 750 ms). Trial ordering was randomized across conditions and the inter-stimulus interval was jittered between 3 and 19 s with a mean interval of 8 s.

Stimuli consisted of colored line drawings that were presented with a written distractor word superimposed (see Figure 1 for examples of the experimental stimuli). Written distractor words were related to the pictures in one of four ways: from the same category (CAT = categorical), a component part of the picture (P-W = part-whole), starting with the same sound (PHONO = phonological), or semantically and phonologically unrelated to the picture (UN = unrelated). Categorically-related distractors were semantically, but not associatively, related to the target image as assessed by the forward cue-to-target strength (FSG, mean FSG = 0.03) generated from the USF Free Association Norms (Nelson et al., 2004). Forward cue-to-target strengths can be determined by presenting individuals with a cue and asking them to generate a related word. The frequency of occurrence of the generated words, calculated across individuals, provides a measure of how strongly the words are associated. The decimal value represents how often a target was generated for a given item (e.g., 0.17 = 17%). The distractors in the part-whole condition were always a component part of the target. Part-whole distractors had significantly higher semantic associations with the target image compared to all other conditions [*F*_(3, 196)_ = 21.66, *p* < 0.001], but overall still maintained a low predictive forward association (mean FSG = 0.17). Phonologically-related distractors shared at least two initial phonemes and letters with the target image (average 2.4, range 2–4), and had no semantic or associative relation to the target (mean FSG = 0). Unrelated distractors contained no semantic or associative relation with the target (mean FSG = 0) and had no phonological or letter-level relation to the target image.

Two hundred color drawings (245 × 350 pixels, 3.4 × 4.9 inches, *N* = 50 per condition) adapted from the Snodgrass and Vanderwart picture set were incorporated (Snodgrass and Vanderwart, 1980; Rossion and Pourtois, 2004). Images depicted common, concrete objects from one of six categories: animals, clothing, fruits/vegetables, household items, landscape items, and tools. Across the four conditions there was no difference in the number of items per object category, χ^2^ (15, *N* = 200) = 12.03, *p* = 0.67 or in the number of living versus non-living items, χ^2^ (3, *N* = 200) = 0.73, *p* = 0.86. Across conditions, pictures did not differ in the number of semantic features (McRae et al., 2005) or visual complexity (Rossion and Pourtois, 2004). The average normed naming consistency was high, 90.88%, and did not differ across conditions (Rossion and Pourtois, 2004). Across conditions, distractor words and picture names were matched for word length, number of syllables, number of phonemes, and imagability using the MRC psycholinguistic database (Coltheart, 1981), and matched for word frequency using the Hal and SUBTL corpora (Balota et al., 2007). Across stimulus categories intended target words did not differ across types of initial phonemes (e.g., plosive, nasal, fricative).

Each trial consisted of a target image and a distractor word presented simultaneously (word–picture SOA = 0, stimulus duration = 750 ms). Words were superimposed in the center of each image using Times New Roman, 20 point font. Participants were asked to name the picture and were instructed to ignore the distractor. Each of five runs (duration = 330 s) began and ended with the presentation of a fixation cross, and a fixation cross was presented between each trial [Inter-stimulus Interval (ISI) range = 3–19 s, *M* = 8 s]. ISIs were optimized with Optseq2 (Dale, 1999) to maximize deconvolution of the hemodynamic response. A jittered ISI and randomized trial order were utilized to minimize participant preparation and anticipation of each stimulus. Trial randomizations were constrained such that no more than two items from one condition appeared in a row. All stimuli were presented using a projector and the CIGAL experimental control program (Voyvodic, 1999; Voyvodic et al., 2011). Participants were instructed to respond with overt verbal responses, but to minimize all other head movement. Practice trials were provided to ensure that participants were comfortable with the procedures and could respond without excessive motion. Overt verbal responses were recorded and filtered during the functional runs using an MR-compatible, fiber optic microphone system (Optoacoustics Ltd., Or-Yehuda, Israel).

### Acquisition of MRI data

Anatomical and functional images were acquired on a 3.0 Tesla GE EXCITE HD whole-body 60 cm bore human scanner equipped with 40 mT/m gradients and a 150 T/m/s slew rate. An eight-channel head coil was used for radio frequency reception (General Electric, Milwaukee Wisconsin, USA). Sagittal T-1 weighted localizer images were acquired and used to define a volume for data collection and high order shimming. The anterior and posterior commissures were identified for slice selection and shimming. A semi-automated high-order shimming program was used to ensure global field homogeneity. T-1 weighted anatomical images were collected with a 3D fSPGR pulse sequence (*TR* = 7.384 ms, *TE* = 2.988 ms, *TI* = 450 ms, FOV = 25.6 cm^2^, flip angle = 12°, voxel size = 1 × 1 × 2 mm, 60 contiguous axial slices). Functional images were acquired with an inverse spiral pulse sequence (*TR* = 1.5 s, *TE* = 30 ms, FOV = 25.6 cm^2^, flip angle = 60°, voxel size = 4 × 4 × 4 mm, 30 contiguous axial slices). Four volumes were acquired at the beginning of each functional run to reach steady state equilibrium; these volumes were deleted and not included in fMRI analysis.

### Data analysis

Overt verbal response accuracies were determined by listening to each filtered audio file and latency was determined using customized MATLAB scripts which calculated the duration between trial and vocalization onsets using an algorithm that calculated deviation from baseline. These latencies were then manually verified through visual and auditory inspections of the speech stream. Verbal responses were counted as errors if the participant failed to respond, read the distractor word, or incorrectly named the picture (e.g., lizard for frog). Outliers were calculated on a subject-by-subject basis and defined as trials that were > 3 SDs from that individual's overall mean latency.

fMRI data were analyzed for quality via a quality assurance tool that quantifies several metrics including Signal-to-Noise (SNR), Signal-Fluctuation-to-Noise (SFNR), motion, and voxel-wise standard deviation measurements (Friedman and Glover, 2006; Glover et al., 2012). Additionally, all data were visually inspected for artifacts and blurring. We used FSL version 4.1.5 and FEAT version 5.98 for preprocessing and for all analyses of functional activations (Smith et al., 2004; Woolrich et al., 2009). Non-brain tissue was removed from participants' functional and anatomical images using the FSL brain extraction tool (Smith, 2002). Pre-processing steps included slice time correction, high-pass filtering, motion correction, co-registration, normalization, and spatial smoothing (FWHM = 8 mm). Functional image data were corrected for slice timing using sinc interpolation to shift each slice in time to the middle of the TR period. Functional data were also high-pass filtered (cut off = 50 s). Functional images were motion-corrected using FSL's MC-FLIRT (FMRIB's Linear Image Registration Tool) using 6 rigid-body transformations (Jenkinson et al., 2002). The average movement in the X, Y, or Z directions was 0.25 mm (range: 0.04–1.94 mm). Thus, none of the included participants exhibited more than 2 mm movement in the X, Y, or Z dimensions. These estimates of motion were included as nuisance covariates in the overall FSL model. Co-registration and normalization steps were completed using FSL's FLIRT, which is an affine registration program (Jenkinson and Smith, 2001; Jenkinson et al., 2002; Greve and Fischl, 2009). Each participant's functional image was co-registered to their own anatomical image and these images were then registered to Montreal Neurological Institute (MNI) standard space using FSL's MNI Avg152 T1 2 × 2 × 2 mm standard brain. The same transformation matrix used to register high resolution anatomical images to MNI was then applied for the functional image to standard-space transformation. fMRIB's improved linear model (FILM) was used to correct for voxel-wise temporal autocorrelation (Woolrich et al., 2001). A double-gamma hemodynamic response function was used to model BOLD signal after each event. Error trials were not included in the fMRI model analyses.

In addition to our motion parameters, we also included several variables as covariates in our FSL analyses because their values may influence the overall pattern of activation. These variables included normed naming latencies for each picture, each participant's naming latency for each trial, and the number of phonemes of distractor words. Naming latencies were included because of significant differences across conditions, and were modeled as a single regressor. Although the number of phonemes in the distractor words was not significant in the overall ANOVA, a *t*-test revealed that there was a marginal difference in the distractor word phonemes when comparing categorical and part-whole conditions, *t*_(198)_ = 1.86, *p* = 0.06.

**Figure 2:**
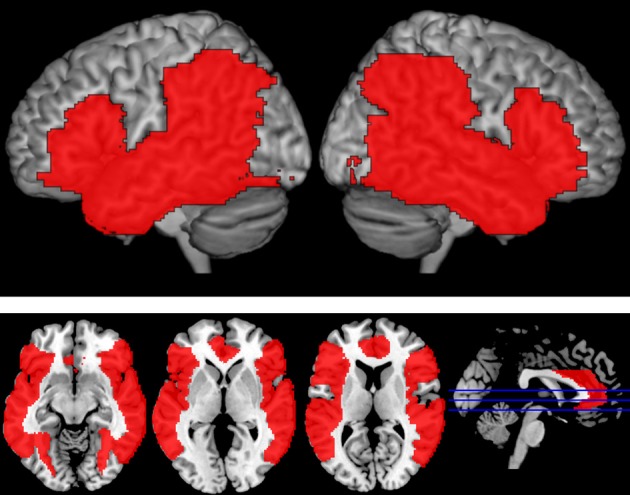
**Language Mask**. Areas in red reflect the fronto-temporo-parietal language mask that was applied at the third level analysis to limit analyses to language-relevant regions. Regions were selected based on previously published reports and included bilateral IFG (BA 44, 45, 47), anterior cingulate, insula, STG, MTG, ITG, fusiform gyri, angular gyri, inferior parietal lobule, and supramarginal gyri.

For each participant, individual runs were combined, and a second-level analysis was performed. An FSL mixed effects model (fMRIB local analysis of mixed effects, FLAME 1 and 2) was used to create a group-level analysis and evaluate activation for each condition (Woolrich et al., 2001; Beckmann et al., 2003). For whole brain analyses, significant activations were determined using a two-step process in which (1) voxels, significant at *p* < 0.001 were identified, and (2) clusters of identified voxels were corrected for multiple comparisons according to Gaussian random fields (GRF) theory (*p* < 0.05, corrected). This process estimates each cluster's significance level by comparing it to the cluster probability threshold, and then only clusters whose estimated significance exceeded the threshold were included in the results (Hayasaka and Nichols, 2003). A fronto-temporo-parietal language mask was applied at the third level analysis to limit analyses to language-relevant regions (e.g., Dronkers et al., 2004; Indefrey and Cutler, 2004; Hickok and Poeppel, 2007; Tyler and Marslen-Wilson, 2008). This approach has been previously adopted by others (e.g., Zhuang et al., 2011; Bozic et al., 2013). This mask included bilateral IFG (BA 44, 45, 47), anterior cingulate, insula, STG, MTG, ITG, fusiform gyri, angular gyri, inferior parietal lobule and supramarginal gyri. An image of the mask can be found in Figure 2. All coordinates are reported in MNI space.

To complement our whole-brain analyses, we also included several anatomically-based regions of interest from the left hemisphere including pars triangularis (BA 45), pars opercularis (BA 44), pars orbitalis (BA 47), anterior STG, posterior STG, middle MTG, angular gyrus, and temporal pole (BA 38). These regions were defined using the Harvard-Oxford Cortical Structural Atlas thresholded at the 25% probability level (Desikan et al., 2006).

## Results

### Behavioral results

The response data from one participant were lost due to a microphone malfunction and additional portions of behavioral data were lost from two subjects due to equipment error (40 trials and 47 trials, distributed equivalently across conditions). The response latencies for the recorded trials were assessed using a one-way ANOVA. Across all naming trials, there was a significant main effect of distractor type on reaction time in a subjects and items analysis [*F*1_(3, 42)_ = 36.3, *p* < 0.001; *F*2_(3, 196)_ = 8.15, *p* < 0.001]. Participants' average reaction times and accuracies are presented in Table 1.Categorically-related trials were responded to significantly slower than all other conditions [P-W; *t*1_(14)_ = 8.70, *p* < 0.001; *t*2_(98)_ = 4.67, *p* < 0.001; PHONO: *t*1_(14)_ = 8.72, *p* < 0.001; *t*2_(98)_ = 2.83, *p* < 0.01; and UN *t*1_(14)_ = 6.66, *p* < 0.001; *t*2_(98)_ = 3.36, *p* < 0.002]. In addition, part-whole trials elicited marginally faster responses than unrelated trials in the subjects analysis [*t*1_(14)_ = 2.04, *p* < 0.061; *t*2_(98)_ = 1.17, *p* < 0.246].

**Table 1:** **Behavioral performance**.

**Condition**	***RT* mean (*SD*)**	***RT* difference**	**Accuracy (*SD*)**
Categorically-related trials	1036 (151) ms	+96 ms^***^	91 (5) %
Part-whole trials	918 (123) ms	−22 ms^**^	97 (3) %^***^
Phonologically-related trials	933 (126) ms	−7 ms	90 (4) %^*^
Unrelated trials	940 (126) ms		93 (5) %

*RT difference is the difference in Reaction Time relative to the unrelated condition*.

*** *significantly different from all other conditions*.

** *significantly different from the categorically-related and unrelated trials*.

* *significantly different from the part-whole and unrelated trials*.

Across all naming trials, there was also a significant main effect of distractor type on accuracy [*F*1_(3, 42)_ = 13.5, *p* < 0.001; *F*2_(3, 196)_ = 3.21, *p* < 0.025]. Responses to part-whole trials were significantly more accurate than all other conditions [CAT: *t*1_(14)_ = 5.57, *p* < 0.001; *t*2_(98)_ = 2.72, *p* < 0.008; PHONO: *t*1_(14)_ = 8.80, *p* < 0.001; *t*2_(98)_ = 2.93, *p* < 0.005; UN: *t*1_(14)_ = 3.40, *p* = 0.004; *t*2_(98)_ = 2.49, *p* < 0.015]. Responses to phonological trials were less accurate than the unrelated trials in the subjects analysis [*t*1_(14)_ = 2.56, *p* < 0.023; *t*2_(98)_ = 1.15, *p* < 0.254], but did not differ from the categorical condition. Verbal fluency scores taken outside of the scanner represent the total verbal fluency scores summed across all categories (F, A, S, and animals). The mean verbal fluency score was 70.0 words (Range 36–103 items, *SD* = 18.5).

### Movement

To assess the influence of movement on overall data quality, we compared the signal to fluctuation noise ratio (SFNR) numbers, an index of fMRI signal stability over time (Glover and Lai, 1998; Kruger and Glover, 2001; Friedman and Glover, 2006), from the task runs to values obtained during a resting state functional run from the same session, during which the participant was not speaking. SFNR values did not significantly differ as a function of overt production [*F*_(5, 90)_ = 1.86, *p* < 0.11]. These results confirm that our instructions and training procedures were effective in minimizing motion and preserving data quality.

### fMRI activation

#### Whole-brain analysis

Our comparisons of interest were those comparing related distractors to the unrelated condition and to each other. The categorical condition did not significantly differ from the unrelated condition. The part-whole condition elicited more activation than the unrelated condition in a cluster in left posterior MTG, which extended into angular and supramarginal gyri (Table 2). Interestingly, there were no significant differences in functional activation between the two semantic conditions: categorical and part-whole, suggesting that these trial types were processed similarly. Because of this, we collapsed across these conditions for subsequent analyses.

**Table 2:** **Part-whole comparisons—areas of activation with sub-peaks**.

	**Volume**			**Peak**		
	**Hemisphere**	**mm^3^**	***Z*-Score**	***X***	***Y***	***Z***
**PART-WHOLE > UNRELATED**						
Middle temporal gyrus	Left	7454	3.30	−58	−54	−6
Angular gyrus			3.20	−54	−54	42
Angular gyrus			2.91	−64	−54	24
Supramarginal gyrus			2.81	−62	−44	−2

**Figure 3:**
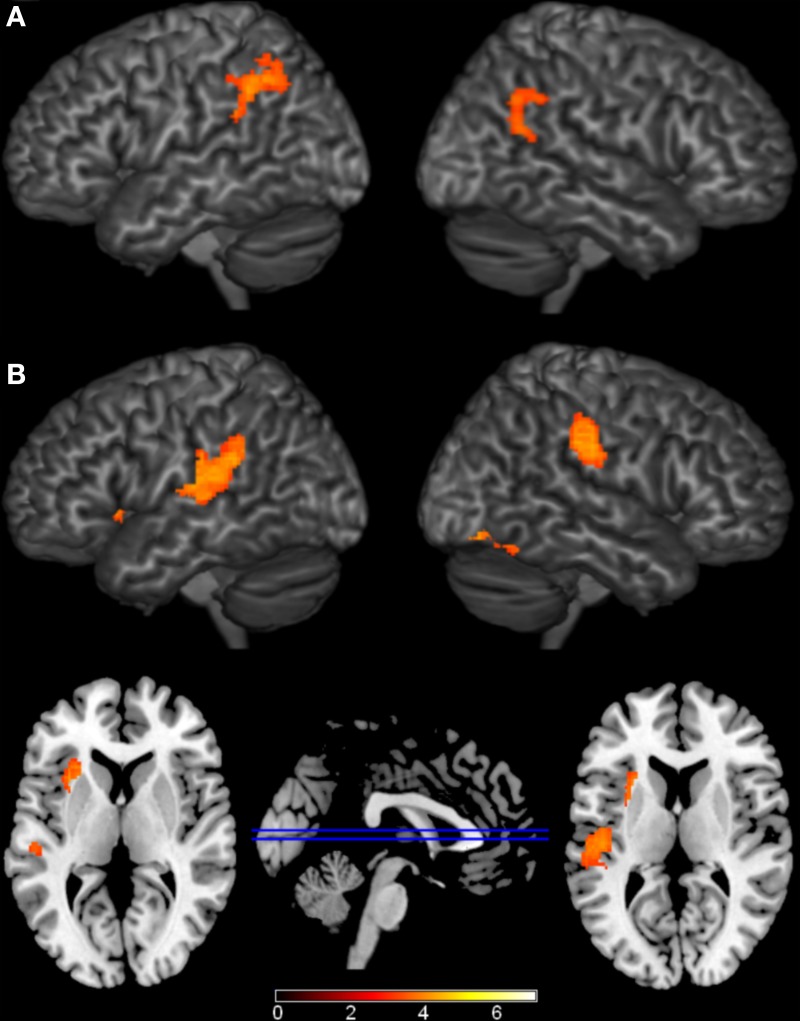
**Brain Activations to the Comparisons with the Phonological Condition**. Areas **(A)** where the phonological condition elicited greater activation than the unrelated condition were found in bilateral parietal cortex. **(B)** Areas where the phonological condition elicited greater activation than the semantic conditions were found in bilateral parietal cortex, left insula, and right occipital cortex. The axial slices highlight the left insula and left Planum Temporale activations. Activations are scaled in terms of significant *Z*-values, and all comparisons are presented at a cluster-corrected threshold of *p* < 0.05.

The largest differences came from comparisons between the phonological condition and the other conditions (Figure 3, Table 3). Compared to unrelated trials, the phonological condition elicited greater activity in bilateral supramarginal and angular gyri. Comparing phonological and semantically related trials, phonological trials elicited greater activation in left insula, left posterior STG which extended into the supramarginal gyrus, right anterior supramarginal gyrus, and a cluster in right fusiform and lingual gyri. Individual comparisons with each of the semantic conditions (e.g., PHON>CAT and PHON > P-W) showed similar regions of activation. There were no conditions that elicited significantly greater activation than the phonological condition.

**Table 3:** **Phonological comparisons—areas of activation with sub-peaks**.

	**Volume**			**Peak**		
	**Hemisphere**	**mm^3^**	***Z*-Score**	***X***	***Y***	***Z***
**PHONOLOGICAL > UNRELATED**						
Supramarginal gyrus	Left	710	4.22	−50	−46	40
Supramarginal gyrus			3.77	−60	−40	28
Angular gyrus			4.14	−52	−54	44
Lateral occipital cortex			3.63	−50	−60	50
Angular gyrus	Right	316	3.79	52	−58	34
Angular gyrus			3.77	56	−58	26
Supramarginal gyrus			3.57	54	−46	36
**PHONOLOGICAL > SEMANTIC**						
Insula	Left	374	4.18	−32	16	4
Insula			4.03	−30	14	8
Insula			4.02	−30	18	−2
Parietal cortex	Left	1542	4.38	−48	−38	26
Central operculum			4.21	−52	−20	12
Planum temporale			4.2	−52	−26	8
Supramarginal gyrus	Right	692	4.33	64	−30	34
Supramarginal gyrus			4.21	62	−22	36
Supramarginal gyrus			4.19	64	−24	42
Occ. Fusiform gyrus	Right	878	5.01	26	−68	−8
Fusiform gyrus			4.42	34	−68	−10
Lateral occipital cortex			4.26	38	−76	−14

**Figure 4:**
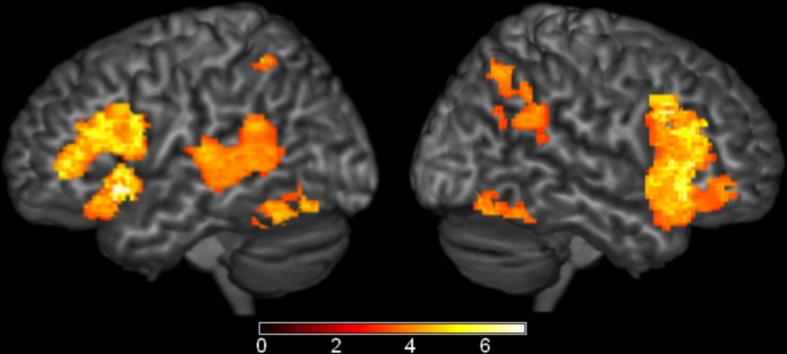
**Brain Activations to Naming Latencies**. Areas where naming latencies to individual trials (i.e., the time it took each participant to name each object) were positively correlated with functional activation are shown in orange and represent areas where increases in activation are correlated with longer naming latencies. Significant activations were found in bilateral inferior frontal gyri (IFG), left insula, left posterior superior temporal gyrus (STG), and bilateral cingulate. Activations are scaled in terms of significant *Z*-values, and all comparisons are presented at a cluster-corrected threshold of *p* < 0.05.

Our model also included a single regressor that modeled participant's naming latencies to individual trials. This comparison revealed several areas where longer naming latencies were associated with increased functional activation (Figure 4, Table 4). These regions included bilateral IFG, left insula, bilateral cingulate gyri, left posterior STG, bilateral superior parietal cortex, right angular gyrus, and bilateral occipital fusiform gyri. The cluster in left posterior STG extended superiorly into the central operculum and supramarginal gyrus, and extended inferiorly into MTG. The patterns of activation to this regressor did not interact with activations to the individual conditions.

**Table 4:** **Positive correlations to naming latency—regions with sub-peaks**.

	**Volume**			**Peak**		
	**Hemisphere**	**mm^3^**	***Z*-Score**	***X***	***Y***	***Z***
Inferior frontal gyrus	Left	3418	6.03	−48	16	14
Frontal pole			5.90	−48	44	6
IFG, pars triangularis			5.78	−50	24	24
IFG, pars triangularis			5.75	−46	36	10
IFG, pars opercularis			5.75	−52	10	26
Inferior frontal gyrus	Right	6286	7.29	40	24	−2
IFG, pars triangularis			6.16	46	28	4
Insula			6.61	38	18	0
Middle frontal gyrus			6.63	46	16	30
Middle frontal gyrus			6.19	56	16	34
Insula	Left	1964	7.32	−44	14	−8
Frontal operculum			6.07	−42	18	0
Orbital frontal cortex			6.61	−34	26	−8
Cingulate gyrus	Bilateral	2870	6.94	−4	38	20
Cingulate gyrus			6.78	8	26	28
Paracingulate gyrus			6.27	2	52	10
Posterior STG	Left	3026	4.92	−56	−22	12
Middle temporal gyrus			4.49	−54	−54	10
Planum temporale			4.76	−58	−24	8
Supramarginal gyrus			4.63	−50	−48	22
Angular gyrus	Right	654	4.34	52	−48	38
Angular gyrus			4.14	52	−46	24
Angular gyrus			4.21	48	−50	28
Supramarginal gyrus			4.27	54	−44	30
Superior parietal lobule	Left	346	4.84	−34	−44	52
Superior parietal lobule			4.17	−36	−54	56
Superior parietal lobule			4.03	−28	−50	52
Superior parietal lobule	Right	470	5.02	32	−62	48
Superior parietal lobule			4.44	32	−54	44
Superior parietal lobule			4.18	28	−54	50
Occipital fusiform gyrus	Left	1764	5.02	−34	−68	−16
Fusiform gyrus			4.78	−34	−70	−10
Inferior temporal gyrus			4.96	−44	−50	−20
Occipital fusiform gyrus	Right	1304	4.73	34	−60	−18
Inferior temporal gyrus			4.7	36	−56	−20
Lateral occipital cortex			4.54	42	−70	−20
Occipital fusiform gyrus			4.52	26	−76	−8

*IFG, Inferior Frontal Gyrus; STG, Superior Temporal Gyrus*.

We also observed significant negative correlations with normed naming latencies in which activation increased as normed naming latencies decreased in bilateral supramarginal gyrus. No other positive or negative correlations were observed with the behavioral covariates included in our FSL model.

### Region of interest analysis

**Figure 5:**
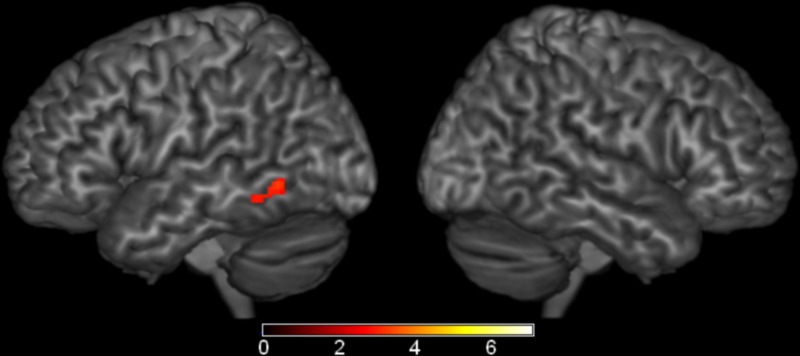
**Brain Activations: Semantic > Phonological**. Areas in red represent the significant voxels within the left middle temporal gyrus region-of-interest where the semantic conditions elicited greater activation than the phonological condition. Activations are scaled in terms of significant *Z*-values, corrected *p* < 0.05.

Although several comparisons of semantic effects did not survive our whole-brain analysis, we wanted to more fully explore weak but reliable effects. To test this, we performed region of interest analyses based on the Harvard-Oxford Cortical Structural Atlas (Desikan et al., 2006) in eight well-established language regions: left posterior STG, posterior MTG, anterior STG, temporal pole, angular gyrus, pars opercularis, pars triangularis, and pars orbitalis. In left posterior MTG (MNI coordinates: −62, −56, −4, Max *Z*-score: 3.85, cluster size: 310 mm^3^), the semantic conditions elicited greater activation than the phonological condition (Figure 5). No significant results were found in any of the other ROIs.

### Correlations with verbal fluency

**Figure 6:**
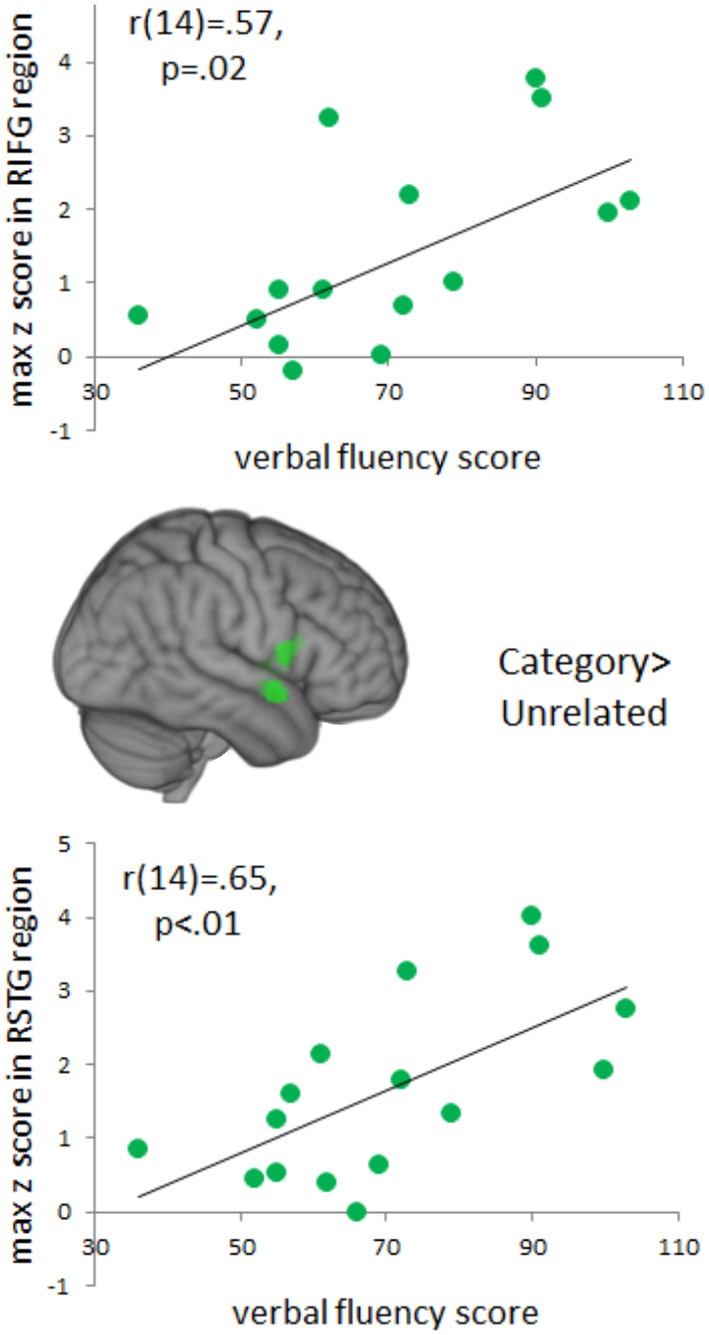
**Brain Activations to Verbal Fluency**. Areas in green indicate voxels where total verbal fluency (F, A, S, and animals) correlated positively with activation to the categorically-related condition. A cluster of significant correlations was found in right anterior, superior temporal gyrus and this cluster extended into right inferior frontal gyrus. Positive correlations represent areas that increased in activation as a function of an individual's total verbal fluency score. Activations are scaled in terms of significant *Z*-values, and all comparisons are presented at a cluster-corrected threshold of *p* < 0.05.

Because individual differences in language production may influence patterns of activation, we investigated possible relationships between verbal fluency and functional activation during naming (Figure 6). Positive correlations between verbal fluency and fMRI activation to categorically-related trials were found in a cluster in right anterior STG (MNI coordinates: 62, 2, −8, max *Z*-score: 3.60, cluster size: 5840 mm^3^). This cluster of activation extended into right IFG and insula (MNI coordinates: 38, 4, −10; max *Z-score:* 3.06) and right pre-central gyrus (MNI coordinates: 60, 8, 8; max *Z*-score: 3.08). There were no significant correlations between verbal fluency and activation to the phonological or part-whole trials.

## Discussion

In the present study, we used fMRI to investigate the influence of linguistically-related distractor words on brain activity during overt language production in a PWI task. Distractors were categorically-related, had part-whole relations, were phonologically-related, or were unrelated. Specifically, we were interested in how different types of semantic distractors influence naming. While categorically-related distractors have been shown to slow naming, behavioral reports indicated that part-whole distractors may provide facilitation (Costa et al., 2005). This is somewhat surprising as both distractors represent additional sources of semantic information and do not provide direct phonological or lexical information about the target. Behaviorally, part-whole trials were responded to faster relative to categorically-related and unrelated trials, and the part-whole condition elicited significantly higher accuracy rates than all other conditions. However, comparisons of fMRI activation between part-whole and categorically-related trials were not significantly different, suggesting a similarity in the neural basis underlying these trials. Comparisons with the phonological condition were also similar across the two semantic conditions. Both semantically-related conditions elicited greater activation than the phonological condition in the left posterior MTG. Theoretical accounts have attributed lexical functions to this region [e.g., as a lexical interface between phonological and semantic information (Hickok and Poeppel, 2007) or for conceptually driven lexical access (Indefrey and Levelt, 2000, 2004)]. Consistent with these accounts, patient studies suggest that damage to this region produces word-level comprehension deficits (Bates et al., 2003; Dronkers et al., 2004). Of note, the similarities in the patterns of fMRI activation for the two semantic conditions may reflect that both stimulus categories involve increased lexical competition between semantically-related items (relative to the phonological condition).

The lack of significant fMRI differences for some of the comparisons may also be due to the robust activation associated with naming latencies overall. Across conditions, naming latencies were positively correlated with activation in several traditional language regions including bilateral IFG, left insula, left posterior MTG/STG, and bilateral superior parietal cortex. This indicated that longer naming latencies were associated with increases in the strength of activation (i.e., z-score) in these regions. This positive correlation most likely reflects increased involvement of these regions corresponding to increased cognitive processing of the stimuli.

Considering the effects of our other linguistically-related distractor condition, the phonologically-related condition elicited greater activation than the semantically-related conditions in left insula, left posterior STG, and bilateral supramarginal gyri. This suggests that these regions are more sensitive to phonological aspects of the stimuli and are relatively insensitive to several types of semantic relations (i.e., part-whole or category membership). Although the temporal resolution of fMRI is too coarse to distinguish among the many stages of language production, these regions are consistent with speech models (Indefrey and Levelt, 2004; Hickok and Poeppel, 2007). The dual stream model of speech includes a parietal-frontal path that translates phonological information, represented posteriorly, to articulatory representations that are required for language production, represented frontally (Hickok and Poeppel, 2007). Their model suggests that an area between temporal and parietal cortex near the Sylvian fissure (Spt) serves as a sensory-motor interface. In contrast, Indefrey and Levelt's model of language production suggests that left posterior STG is involved in phonological code retrieval and self-monitoring (Indefrey and Levelt, 2004; Indefrey, 2011). Involvement of posterior STG and inferior parietal lobe in phonological processes is consistent with patient studies showing damage to this region produces word repetition deficits, consistent with a role in verbal working memory (Dronkers et al., 2004). Other imaging studies have demonstrated that inferior parietal cortex, especially left supramarginal gyrus, is sensitive to phonological demands (Church et al., 2011) and implicit phonological priming (Wilson et al., 2011).

The frontal component of our phonological regions (left insula) has been hypothesized to contribute to phonological code retrieval (Indefrey and Levelt, 2004; Indefrey, 2011) and to be a component of the articulatory network (Hickok and Poeppel, 2007). Consistent with these interpretations, lesion studies have linked the insula to fluency deficits (Dronkers, 1996; Bates et al., 2003). Moreover, decreases in functional activation (Shafto et al., 2010) and declines in structural integrity (Shafto et al., 2007; Stamatakis et al., 2011) within this region have been linked to failed phonological retrieval, as the in tip-of-the-tongue phenomenon among healthy older adults.

Of note, we observed increases in activation to the phonological condition concurrent with behavioral facilitation. The direction of this effect, while consistent with Abel et al. (2009, 2012), is opposite from what would be predicted from traditional priming experiments and is in contrast to results from other PWI experiments (De Zubicaray et al., 2002; De Zubicaray and Mcmahon, 2009). One hypothesis is that the enhanced activation we observed for phonological trials in left insula, posterior STG, and bilateral inferior parietal cortex could reflect the dual activation of the target and distractor. That is, a strong phonological cue combined with the target may create a more focused, and salient stimulus. Others have argued that mechanisms underlying the PWI task may involve both facilitation due to priming-related mechanisms and increases in activation due to processing multiple different stimuli (Abel et al., 2012).

This discrepancy in terms of the direction of the phonological effects cannot be explained by differences in the modality of the distractor or SOA, as these factors were the same across studies. One design factor that may have influenced the differences in results is stimulus repetition. Unlike the present study and Abel et al. (2009), participants in the de Zubicaray et al. studies practiced naming the pictures before scanning began and the same stimuli were repeated across conditions. This design has the benefit of controlling for perceptual features and names of the pictures across conditions, but also entails participants naming the pictures multiple times and effects of stimulus repetition could interact with distractor effects. De Zubicaray et al. (2002) argue that phonologically-related distractor trials may elicit a facilitatory effect via priming in conjunction with increased interference relative to unrelated trials^2^.

We also wanted to assess the potential influence of individual differences in language production on patterns of functional activation. Our results indicated that individual differences in verbal fluency assessed behaviorally outside of the scanner positively correlated with fMRI activation to categorically-related trials relative to unrelated trials in right anterior superior temporal regions. Although it may seem surprising to see effects within the right hemisphere, research suggests that the right hemisphere also supports language in important but perhaps more subtle ways. Studies support right hemisphere involvement in a variety of language-related tasks including figurative language processing (Bottini et al., 1994; Ahrens et al., 2007; Chen et al., 2008; Diaz et al., 2011), discourse (Ferstl and Von Cramon, 2001; Kuperberg et al., 2006; Ferstl et al., 2008; Price, 2010; Diaz and Hogstrom, 2011), conceptual representation and specifically in combining concepts into larger meaningful units (e.g., Graves et al., 2010). Of particular relevance to the PWI task, prior studies using behavioral, ERP, and neuropsychological methods have suggested that the right hemisphere may play a particular role in the processing of non-associative semantic relationships like category membership (e.g., Jung-Beeman and Chiarello, 1998; Federmeier et al., 2008). Consistent with this, the current study and previous PWI experiments have reported activation within the right hemisphere (Abel et al., 2009; De Zubicaray and Mcmahon, 2009). Additionally, patient research has indicated that individuals with right hemisphere damage showed reduced priming for categorically-related words, even though associative priming was preserved (Hagoort et al., 1996). Moreover, recent structural (Catani et al., 2007) and functional (Van Ettinger-Veenstra et al., 2010) imaging studies suggest that individual differences in the right hemisphere are positively correlated with language performance. These correlations highlight the importance of examining relevant individual differences and suggest that activation within the right hemisphere may be particularly sensitive to individual differences.

## Summary

Using a PWI task, we examined the influence of four types of distractors on picture naming: categorically-related, part-whole, phonologically-related, and unrelated distractors. We observed similar patterns of activation for categorically-related and part-whole trials which may reflect the increased lexical competition that both conditions afford. We also observed increased activation to the phonological condition relative to both semantic conditions in left insula, posterior STG, and bilateral inferior parietal cortex. This both confirms the involvement of these regions during phonological encoding and retrieval, and suggests a relative insensitivity of these regions to a variety of semantic relations.

## Author contributions

Michele T. Diaz and Larson J. Hogstrom designed the experiment. Michele T. Diaz, Larson J. Hogstrom, and James T. Voyvodic conducted the research. Michele T. Diaz, Jie Zhuang, and C. Christine Camblin analyzed data. Michele T. Diaz, Larson J. Hogstrom, Jie Zhuang, Micah A. Johnson, and C. Christine Camblin wrote the paper.

### Conflict of interest statement

The authors declare that the research was conducted in the absence of any commercial or financial relationships that could be construed as a potential conflict of interest.

## Footnotes

^1^We place the level of facilitation at the phonological level, even though many studies have used written distractors. This is because (1) orthography strongly activates phonology and (2) production requires phonological as opposed to orthographic retrieval.

^2^According to this hypothesis, the priming effect has a larger impact on behavioral measures resulting in the facilitation usually found for phonologically-related distractor trials.
