# Supplementary material for: Written distractor words influence brain activity during overt picture naming
Source: Front Hum Neurosci. 2014 Mar 24;8:167. doi: 10.3389/fnhum.2014.00167 (PMC3970014; doi:10.3389/fnhum.2014.00167)
Supplement: Supplementary file 1 [file DataSheet1.DOCX]

Supplemental Table 1: Stimuli

Stimulus Distractor Target Initial IPA

Category Word Word Phoneme Category

Categorical hip arm ä vowel

shed barn b plosive

racket bat b plosive

mosquito bee b plosive

dragonfly beetle b plosive

plate bowl b plosive

muffin cake k plosive

squash carrot k plosive

rope chain ch fricative

raspberry cherry ch fricative

blazer coat k plosive

ottoman couch k plosive

gate door d plosive

latch doorknob d plosive

shelf dresser d plosive

cymbal drum d plosive

hawk eagle ē vowel

saxophone flute f fricative

tuba French horn f fricative

mug glass g plosive

diamond heart h whisper

Supplemental Table 1: Stimuli (continued)

Stimulus Distractor Target Initial IPA

Category Word Word Phoneme Category

Categorical pad helmet h whisper

ox horse h whisper

cheetah lion l liquids

crab lobster l liquids

canyon mountain m nasal

pepper mushroom m nasal

bracelet necklace n nasal

chin nose n nasal

lime orange ä vowel

almond peanut p plosive

mango pear p plosive

turnip potato p plosive

washer refrigerator r liquids

compass ruler r liquids

bolt screw sk fricative

hammer screwdriver sk fricative

walrus seal s fricative

bull sheep sh fricative

possum skunk sk fricative

counter table t plosive

radio television t plosive

sign traffic light t plosive

Supplemental Table 1: Stimuli (continued)

Stimulus Distractor Target Initial IPA

Category Word Word Phoneme Category

Categorical grass tree t plosive

lizard turtle t plosive

jug vase v fricative

bass violin v fricative

cup wine glass w glide

saw wrench r liquids

antelope zebra z fricative

Part-Whole cockpit airplane e vowel

blade axe a vowel

rubber balloon b plosive

peel banana b plosive

sheet bed b plosive

gear bicycle b plosive

wing bird b plosive

handle broom b plosive

wick candle k plosive

steeple church ch fricative

antlers deer d plosive

beak duck d plosive

pupil eye ī vowel

stake fence f fricative

knuckle finger f fricative

Supplemental Table 1: Stimuli (continued)

Stimulus Distractor Target Initial IPA

Category Word Word Phoneme Category

Part-Whole petal flower f fricative

heel foot f fricative

neck giraffe j fricative

frames glasses g plosive

string guitar g plosive

follicle hair h whisper

wire hanger h whisper

brim hat h whisper

fabric jacket j fricative

pouch kangaroo k plosive

shade lamp l liquids

stem leaf l liquids

thigh leg l liquids

spots leopard l liquids

feather peacock p plosive

eraser pencil p plosive

snout pig p plosive

strap pocketbook p plosive

horn rhinoceros r liquids

buckle roller skate r liquids

cheese sandwich s fricative

cuff shirt sh fricative

Supplemental Table 1: Stimuli (continued)

Stimulus Distractor Target Initial IPA

Category Word Word Phoneme Category

Part-Whole lace shoe sh fricative

hem skirt sk fricative

coal snowman s fricative

tail squirrel sk fricative

dial stove s fricative

zipper suitcase s fricative

wool sweater s fricative

cord telephone t plosive

bristles toothbrush t plosive

boxcar train t plosive

seed watermelon w glide

tire wheel w glide

curtain window w glide

Phonological apparel apple a vowel

artist artichoke ä vowel

banner barrel b plosive

berry bear b plosive

booth boot b plosive

bumper butterfly b plosive

butter button b plosive

camera camel k plosive

candy cannon k plosive

Supplemental Table 1: Stimuli (continued)

Stimulus Distractor Target Initial IPA

Category Word Word Phoneme Category

Phonological cap caterpillar k plosive

cellar celery k plosive

chimp chicken ch fricative

chips chisel ch fricative

closet clock k plosive

coconut comb k plosive

desert desk d plosive

dolphin doll d plosive

dock donkey d plosive

elbow elephant e vowel

fig fish f fricative

flask flag f fricative

gorge gorilla g plosive

harness harp h whisper

lasso ladder l liquids

lens lemon l liquids

letter lettuce l liquids

minister mitten m nasal

mouth motorcycle m nasal

motel mouse m nasal

nugget nut n nasal

panda pants p plosive

Supplemental Table 1: Stimuli (continued)

Stimulus Distractor Target Initial IPA

Category Word Word Phoneme Category

Phonological pebble penguin p plosive

pie pipe p plosive

plunger plug p plosive

pond pot p plosive

puppet pumpkin p plosive

rag rabbit r liquids

room rooster r liquids

spine spider s fricative

stoop stool s fricative

subway sun s fricative

swamp swan s fricative

swimmer swing s fricative

tile tie t plosive

timer tiger t plosive

tobacco toaster t plosive

toad tomato t plosive

truffle trumpet t plosive

whip whistle w glide

willow windmill w glide

Unrelated egg accordion ə vowel

dentist alligator a vowel

skin anchor a vowel

Supplemental Table 1: Stimuli (continued)

Stimulus Distractor Target Initial IPA

Category Word Word Phoneme Category

Unrelated mineral asparagus ə vowel

stage ball b plosive

canoe basket b plosive

cork bell b plosive

wolf blouse b plosive

cube bottle b plosive

walnut bow b plosive

ocean bread b plosive

penny bus b plosive

river cat k plosive

shovel chair ch fricative

garlic cigarette s fricative

pool cloud k plosive

vacuum clown k plosive

tent corn k plosive

tea crown k plosive

burner dog d plosive

rust dress d plosive

castle ear i vowel

megaphone envelope e vowel

wheat fly f fricative

speaker football f fricative

Supplemental Table 1: Stimuli (continued)

Stimulus Distractor Target Initial IPA

Category Word Word Phoneme Category

Unrelated mortar frog f fricative

clip goat g plosive

knee grapes g plosive

paddle grasshopper g plosive

latch iron ī vowel

pyramid kettle k plosive

bead knife k plosive

cattle lock l liquids

motor nail n nasal

ham needle n nasal

leech onion ə vowel

bean ostrich ä vowel

square owl au̇ vowel

yacht paintbrush p plosive

caribou pliers p plosive

drill raccoon r liquids

roller sailboat s fricative

tractor scissors s fricative

tooth sled s fricative

coupon snail s fricative

wood snake s fricative

bubble strawberry s fricative

Supplemental Table 1: Stimuli (continued)

Stimulus Distractor Target Initial IPA

Category Word Word Phoneme Category

Unrelated scaffold toe t plosive

pea vest v fricative

tomb wagon w glide
